# Non-engineered and Engineered Adult Neurogenesis in Mammalian Brains

**DOI:** 10.3389/fnins.2019.00131

**Published:** 2019-02-21

**Authors:** Wenliang Lei, Wen Li, Longjiao Ge, Gong Chen

**Affiliations:** ^1^Guangdong-Hong Kong-Macau Institute of CNS Regeneration, Jinan University, Guangzhou, China; ^2^Kunming Institute of Zoology, Chinese Academy of Sciences, Kunming, China; ^3^Department of Biology, Huck Institutes of the Life Sciences, Pennsylvania State University, University Park, PA, United States

**Keywords:** neurogenesis, *in vivo* reprogramming, glia-to-neuron conversion, brain repair, neuron, astrocyte

## Abstract

Adult neurogenesis has been extensively studied in rodent animals, with distinct niches found in the hippocampus and subventricular zone (SVZ). In non-human primates and human postmortem samples, there has been heated debate regarding adult neurogenesis, but it is largely agreed that the rate of adult neurogenesis is much reduced comparing to rodents. The limited adult neurogenesis may partly explain why human brains do not have self-repair capability after injury or disease. A new technology called “*in vivo* cell conversion” has been invented to convert brain internal glial cells in the injury areas directly into functional new neurons to replenish the lost neurons. Because glial cells are abundant throughout the brain and spinal cord, such engineered glia-to-neuron conversion technology can be applied throughout the central nervous system (CNS) to regenerate new neurons. Thus, compared to cell transplantation or the non-engineered adult neurogenesis, *in vivo* engineered neuroregeneration technology can provide a large number of functional new neurons *in situ* to repair damaged brain and spinal cord.

## Introduction

Human brain has billions of neurons and even more number of glial cells. Neurons cannot divide and therefore they do not self-regenerate after injury, but glial cells can proliferate upon injury or disease. Human neurons inside the brain are believed to live most of our whole life and cannot be replaced once being damaged or degenerated. It is perhaps due to this doctrine that the first finding of adult neurogenesis in rodent animals in 1960’s (Altman and Das, 1965) was not well recognized until decades later. Nevertheless, the identification of newborn neurons in human postmortem brains (Eriksson et al., 1998) did trigger greater enthusiasm in searching for internal neural stem cells for brain repair. After two decades of research, it is found that adult neurogenesis in mammalian brains is largely restricted to a few discrete niches such as the hippocampus and the subventricular zone (Ming and Song, 2011). Some studies suggest a possibility of neuroprogenitor cells migrating toward injury sites to differentiate into neurons, but further lineage tracing studies demonstrate that the *in vivo* differentiated cells are mainly glial cells rather than neurons (Buffo et al., 2008; Faiz et al., 2015). Thus, a consensus is that while mammalian brains have adult neurogenesis, the number of newborn neurons is likely limited in a few regions, making it difficult to repair damaged brains.

To overcome the limitation of endogenous neurogenesis, scientists transplanted external stem cells into the brain or spinal cord in order to regenerate new neurons in any injured areas regardless whether it is close to a neurogenic niche or not. This typically involves *in vitro* cell cultures to expand the stem cells or even partially differentiate the stem cells toward some fate-determined neuroprogenitor cells. This *in vitro* cell culture followed with *in vivo* transplantation is referred here as “*in vitro* engineered neurogenesis.” Recently, a new technology called “*in vivo* cell conversion technology” has been invented by making use of internal glial cells to generate new neurons (Niu et al., 2013; Torper et al., 2013; Guo et al., 2014; Su et al., 2014), eliminating the *in vitro* steps of cell culture and the following transplantation procedures. Because glial cells are distributed throughout the brain and spinal cord, this *in vivo* cell conversion technology can be applied anywhere in the central nervous system (CNS), and referred here as “*in vivo* engineered neurogenesis.” While still in its early stage, this *in vivo* cell conversion technology already shows some promise in brain and spinal cord repair. This review will focus on this new emerging technology of *in vivo* engineered neurogenesis and compare with *in vitro* engineered neurogenesis as well as the endogenous neurogenesis in the hippocampus and subventricular zone.

## Endogenous Neurogenesis

### Adult Neurogenesis in Rodents

Half century ago, researchers provided the first evidence of neurogenesis in postnatal rats (Altman and Das, 1965). It took more than a decade to confirm neurogenesis in adult rats (Kaplan and Hinds, 1977; Bayer et al., 1982) and adult mice (Reynolds and Weiss, 1992; Lois and Alvarez-Buylla, 1993; Lois and Alvarez-Buylla, 1994). Intriguingly, the magnitude of adult-generated neurons in the rat dentate gyrus is elevated significantly by the hippocampal dependent associative learning tasks (Gould et al., 1999a). High dose BrdU injection suggested a large pool of new neurons being added in the dentate gyrus of young adult rats every day (Cameron and McKay, 2001). While the hippocampus and subventricular zone (SVZ) are two well-documented neurogenic niches in adult rodents, a small number of BrdU-labeled neurons were also found in other brain regions (Zhao et al., 2003; Dayer et al., 2005; Kokoeva et al., 2005). The adult neurogenesis can be regulated by a variety of signaling pathways including the Noggin/Bone Morphogenetic Protein (BMP) signaling (Lim et al., 2000), Wnt signaling (Kuwabara et al., 2009), the activity-induced immediate early gene Gadd45b (Ma et al., 2009), a brain-enriched microRNA miR-124 (Cheng et al., 2009), methyl-CpG–binding protein 2 (MeCP2) (Szulwach et al., 2010), and other factors (Braun and Jessberger, 2014; Goncalves et al., 2016). Furthermore, many SGZ newborn neurons undergo apoptosis during the first few days of life, leaving a small amount surviving in the adult brain (Sierra et al., 2010). Those surviving newborn neurons in adult rodents are believed to contribute to many brain functions including but not limited to olfactory- and hippocampus-dependent learning and memory (Aimone et al., 2006, 2011; Akers et al., 2014). Moreover, adult neurogenesis may play crucial roles in other cognitive functions like spatial and object recognition and fear conditioning, and correlate with disease processes such as major depression, epilepsy and major affective disorders (Cameron and Glover, 2015; Lepousez et al., 2015; Sailor et al., 2017). Recent studies using single-cell RNA-seq successfully characterized the genome-wide molecular signatures of adult NSCs in mouse dentate gyrus (Shin et al., 2015), and revealed that dormant ependymal NSCs are located in the lateral and the fourth ventricles (Luo et al., 2015). Over the past several decades, we have gained much advanced understandings on the endogenous adult neurogenesis in rodent animals (for more comprehensive review, please see Ming and Song, 2011; Goncalves et al., 2016; Lim and Alvarez-Buylla, 2016).

### Adult Neurogenesis in Non-human Primates

In the 1980s, Rakic and colleagues using ^3^H-thymidine autoradiographic technique found newborn neurons in the SGZ of rhesus monkeys before and after birth, but could not detect newborn neurons after puberty (Rakic, 1985; Eckenhoff and Rakic, 1988). Later, via BrdU labeling method, Kornack and Rakic found some newborn neurons in the GCL in adult macaque monkeys, but the relative rate of neurogenesis is approximately 10 times less than the rodents (Kornack and Rakic, 1999). Gould and Tanapat (1999), Gould et al. (1999b) also demonstrated adult neurogenesis in the hippocampus and SVZ of both marmoset monkeys and Old-World monkeys. The rostral migratory stream (RMS), a migratory route in the brain along which neuronal precursors originated in the subventricular zone migrate to reach the olfactory bulb, has been reported in the adult monkey, similar to that in rodents (Kornack and Rakic, 2001b; Sawamoto et al., 2011). Moreover, newborn neurons are also reported in the amygdala, piriform cortex, and adjoining inferior temporal cortex in adult primates (Bernier et al., 2002), but the adult neurogenesis in monkey neocortex remains controversial (Gould and Tanapat, 1999; Gould et al., 2001; Kornack and Rakic, 2001a). Although there are only limited studies in non-human primates, there seems to be a small number of newborn neurons detectable in adult primates, but the rate of neurogenesis is greatly reduced comparing to adult rodents (Sorrells et al., 2018).

### Adult Neurogenesis in Humans

It is even more controversial regarding adult neurogenesis in humans. The first evidence of human adult neurogenesis was provided in 1998, when Gage and colleagues discovered the presence of BrdU-labeled neurons in the hippocampus of cancer patients receiving BrdU for diagnosis purpose (Eriksson et al., 1998). Since then, increasing studies have documented the extent of adult neurogenesis in humans, but the overall rate of neurogenesis in humans sharply declines after birth and are nearly absent in adulthood (Sanai et al., 2011; Sorrells et al., 2018). Curtis et al. (2007) examined the brains of deceased cancer patients, who had previously been injected with BrdU, and found BrdU-positive cells in the patients’ olfactory bulbs, suggesting that RMS might exist in adult humans. However, this was challenged by Sanai et al. (2011), who used a proliferation marker Ki67 and the immature neuronal marker DCX to show that SVZ neurogenesis and migration along the RMS ceased by 2 years after birth in humans. More recent studies demonstrated that the neuroblasts generated in the human SVZ seldom migrate to the olfactory bulb, but rather to the nearby striatum (Bergmann et al., 2012; Ernst et al., 2014).

Hippocampal neurogenesis in adult human brains is also highly debated. Carbon birth-dating method implicated that hundreds of newborn neurons might be added to the adult human dentate gyrus (DG) per day with a modest decline during aging (Spalding et al., 2013), but immunocytochemistry analysis found an age-related sharp decrease of proliferating neuroblasts and putative newborn neurons (Knoth et al., 2010; Dennis et al., 2016). Recently, two prominent studies with similar data but opposite conclusions have re-ignited the debate. Sorrells et al. (2018) found that human hippocampal neurogenesis dropped sharply in children (7–13 years old) and to almost undetectable level in adults (18–77 years), whereas Boldrini et al. (2018) found existence of newborn neurons not only in young age samples but also from old patient samples (14–79 years old). Both studies used similar markers for proliferating neuroprogenitors and immature neurons but drew opposite conclusions, although the actual data was quite similar—a few thousands of newborn neurons in the adult human hippocampus. Given billions of neurons in the human brain, whether these thousands of neurons can carry out a specific function remains a question to be answered.

**FIGURE 1:**
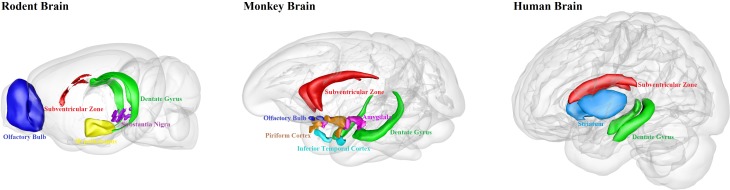
Schematic illustration of adult neurogenesis sites in the brains of rodent, monkey and human. Neurogenesis takes place throughout life in the hippocampal dentate gyrus and the subventricular zone in rodent, monkey and human brains. Newborn cells from the subventricular zone migrate to the olfactory bulb in rodents and monkeys, but maybe not in humans. Adult neurogenesis has also been found in some other brain regions, such as hypothalamus and substantia nigra in rodents; amygdala, piriform cortex and inferior temporal cortex in monkeys; and striatum in humans. This figure is inspired by the Scalable Brain Atlas website and its 3-D Composer.

We have summarized the well-documented sites of adult neurogenesis in mammalian brains (Figure 1). Human brain has limited adult neurogenesis sites.

When comparing adult neurogenesis among rodents, primates, and humans, one can find that hippocampal neurogenesis is conserved among mammals, but there are still differences between rodents and primates. The distribution of cell population and the cytoarchitecture of the SVZ is different between rodents and primates (Sanai et al., 2004, 2011). In primates, the SVZ have a three-layer organization, with an ependymal layer, a hypocellular gap layer, and an astrocyte ribbon layer; in contrast, adult mice do not have the hypocellular gap layer, and the mouse SVZ is composed of neuroblasts, glial cells, and precursor cells (Brus et al., 2013). Moreover, there are large differences between humans and other mammals in the output of new neurons from the SVZ. In rodents and non-human primates, neuroblasts coming from the SVZ migrate to the OB. However, humans do not have obvious addition of new neurons in the OB after 2 years of birth (Bergmann et al., 2012). Lastly, only a few new neuroblasts appear to be produced in the SVZ in humans (Ernst and Frisen, 2015). In humans dentate gyrus, most of the neurons might have been replaced after birth, compared to only ∼10% replaced in mice at birth (Spalding et al., 2013). On the other hand, the maturation of newborn neurons takes much longer time in primates, making the adult-born neurons more difficult to survive than the neurons born during embryonic development (Kohler et al., 2011). Together, we should be cautious in extrapolating the findings on adult neurogenesis from rodents to primates, and in particular not to imply any direct clinical applications for humans.

## *In Vitro* Engineered Neurogenesis

Besides relying upon endogenous neural stem cells to generate new neurons, transplantation of external cells into the brain and spinal cord has been explored as an alternative approach for neuroregeneration. The major external cell source for transplantation includes embryonic stem cells (ESCs), mesenchymal stem cells (MSCs), induced pluripotent stem cells (iPSCs), organoids, and other *in vitro* differentiated or *trans*-differentiated cells (Yang et al., 2011; Trounson and McDonald, 2015). Transplantation of external cells into the brain has faced tremendous technical challenges such as immunorejection, long-term survival and functional integration, as well as tumorigenesis (Trounson and McDonald, 2015; Goldman, 2016). This review will briefly summarize the recent progress on *in vitro* engineered neurogenesis, putting more emphasis on *in vivo* engineered neurogenesis in the next section.

### ESC/iPSC Technology

Embryonic stem cells are expandable *in vitro* and able to differentiate into functional neuronal subtypes in defined medium. Their efficacy to alleviate neurological abnormalities has been proven in a number of animal models, and clinical trials using ESC-derived neural cells have been initiated to treat several neurological disorders such as macular degeneration, Parkinson’s disease and spinal cord injury (Trounson and McDonald, 2015). iPSCs are reprogrammed from somatic cells (Takahashi and Yamanaka, 2006; Takahashi et al., 2007) with defined factors or small molecules, and can be differentiated into a variety of neuronal subtypes but circumvent the immunorejection and ethical issues faced by ESCs (Martin, 2017). The first clinical trial using iPSC-derived retinal pigment epithelium was conducted in Japan on a patient suffering from age-related macular degeneration (Cyranoski, 2014). Recently, new clinical trial using iPSC-derived dopaminergic neurons to treat Parkinson’s patients has been announced in Japan after successful preclinical studies in monkeys (Kikuchi et al., 2017). Engrafted stem cell-derived neurons expressing channel rhodopsin or DREADDs (designer receptors exclusively activated by designer drugs) can be activated in an optical or chemical-controllable fashion to modulate mouse motor functions (Bryson et al., 2014; Steinbeck et al., 2015; Chen et al., 2016), suggesting that some of the transplanted cells have integrated into the preexisting circuits. On the other hand, it should be cautioned that for wide-spreading neurodegenerative disorders such as Alzheimer’s disease, it is more difficult for cell transplantation to reach every corner of the degenerating brain.

### *In vitro* Neuronal *Trans*-Differentiation

The idea of using transcriptional factors to reprogram somatic cells into stem cells (Takahashi and Yamanaka, 2006; Takahashi et al., 2007) has inspired a new field called “cell *trans*-differentiation,” where almost any cell type can be reprogrammed or *trans*-differentiated into a different cell type (Vierbuchen et al., 2010; Tsunemoto et al., 2015, 2018). Vierbuchen et al. (2010) first reported a groundbreaking work in converting skin fibroblast cells into induced neurons (iNs) using three transcription factors Ascl1, Brn2, and Myt1l. Since then, various subtypes of iNs including glutamatergic, GABAergic, dopaminergic, motor neurons, and retinal neurons have been successfully *trans*-differentiated from a number of initial somatic cells such as fibroblasts, hepatocytes, pericytes, astrocytes, and peripheral T cells (Heinrich et al., 2010; Addis et al., 2011; Caiazzo et al., 2011; Marro et al., 2011; Pfisterer et al., 2011; Son et al., 2011; Yoo et al., 2011; Giorgetti et al., 2012; Karow et al., 2012; Ladewig et al., 2012; Liu et al., 2013; Xue et al., 2013; Victor et al., 2014; Colasante et al., 2015; Li et al., 2015; Zhang et al., 2015; Tanabe et al., 2018). iNs bypass the process of iPSCs and have minimal tumorigenic risk. In terms of cell replacement, there are already a few reports on the transplantation of iNs *in vivo* (Kim et al., 2011; Son et al., 2011; Colasante et al., 2015; Hu et al., 2015; Mertens et al., 2015), but more comprehensive evaluations are required before clinical translation. One interesting study found that iNs converted from fibroblasts of elder donor preserved the cellular signature of aging, suggesting that iNs may be a better *in vitro* model than iPSCs to study aging (Mertens et al., 2015).

### Brain Organoids

Neuroprogenitors differentiated from iPSCs can self-organize *in vitro* to form 3D complex structures containing areas resembling diverse regions of the brain (Di Lullo and Kriegstein, 2017; Amin and Pasca, 2018; Pasca, 2018). These structures are named “brain organoids.” Different region-specific organoids can fuse together to develop brain assembloids (Di Lullo and Kriegstein, 2017; Pasca, 2018). Brain organoids hold promise to accelerate our understanding of human brain development and brain disorders due to their several distinct advantages. First, human brain organoids display specific features of human brain development that are absent in rodent models, such as the presence of outer radial glial cells (Di Lullo and Kriegstein, 2017). Second, brain organoids generated from patient-derived iPSCs can better recapitulate the structural and functional complexity of the human brain than the 2D monolayer systems, and thus more precisely modeling neurological disorders for mechanistic studies or for drug screening (Amin and Pasca, 2018). Third, brain organoids can be manipulated with genome editing techniques using CRISPR-Cas9 before being transplanted into animals to further study *in vivo* development (Pasca, 2018). On the other hand, brain organoids also have limitations. They are typically in an early phase of development, and thus may fail to model some later developmental events or late-onset disease phenotypes (Di Lullo and Kriegstein, 2017). Additionally, the extent of diverse cell types in the organoids so far has not been fully validated and the reproducibility of compositions is poor (Amin and Pasca, 2018). Moreover, the transcriptional identity of some cells in the organoids is not closely correlated to an *in vivo* counterpart (Di Lullo and Kriegstein, 2017). Finally, brain organoids currently lack some of the cell types such as endothelial cells and microglia, which are involved in normal brain development and circuit function (Di Lullo and Kriegstein, 2017; Pasca, 2018). Future works are required to overcome these limitations.

## *In Vivo* Engineered Neurogenesis

### Engineered Adult Neurogenesis Through Glia-to-Neuron Conversion

To overcome the limitation of endogenous neurogenesis and the disadvantages of external cell transplantation, *in vivo* engineered neurogenesis has been brought to the front stage in recent years by making use of internal glial cells to generate neurons through *in vivo* glia-to-neuron conversion technology. Importantly, unlike the endogenous neurogenesis that is restricted in certain niches, *in vivo* engineered neurogenesis can be induced in any places throughout the brain and spinal cord (Li and Chen, 2016). Moreover, different subtypes of neurons have been regenerated in different brain regions as discussed below.

### *In vivo* Reprogramming Glial Cells Into Glutamatergic Neurons

Neuroprogenitor cells (NPCs) are known to express certain glial markers such as GFAP, and astrocytes after injury can express certain NPC markers such as Nestin and Sox2. Such observation once confused some scientists that glial cells might be a kind of NPC-like cells, but lineage-tracing studies found that glial cells rarely generate neurons *in vivo* (Buffo et al., 2008; Faiz et al., 2015). However, overexpression of a mutant transcription factor Olig2 in the stab-wound mouse cortex (Buffo et al., 2005) or overexpressing neurogenin 2 (Ngn2) and Ascl1 (Mash1) in injured spinal cord (Ohori et al., 2006) both generated immature neurons, suggesting that it might be possible to directly engineer adult neurogenesis *in vivo*. At that time, it was not clear whether new neurons were generated from NPCs or glial cells, because they share some common properties (Gotz et al., 2015). Later, Berninger et al. (2007) demonstrated that single transcription factor neurogenin-2 (Ngn2) could convert cultured postnatal cortical astroglia into glutamatergic neurons *in vitro*. After repeating the conversion of cultured astrocytes into neurons induced by Ngn2 in cell cultures, we further tested whether it is possible to directly convert astrocytes into neurons inside mouse brains *in vivo*. It was a bit disappointing that injecting retroviruses expressing Ngn2 alone in adult mouse cortex *in vivo* yielded very few newborn neurons. However, after replacing Ngn2 with a different transcription factor NeuroD1, we observed high efficiency conversion (90%) of reactive astrocytes into glutamatergic neurons in adult mouse cortex (Guo et al., 2014). More importantly, unlike immature neurons reported earlier, NeuroD1-converted neurons are fully functional, showing repetitive action potentials and robust synaptic responses that are similar to their neighboring neurons (Guo et al., 2014). NeuroD1 can also convert NG2 cells into mostly glutamatergic neurons, with a small population being GABAergic neurons (Guo et al., 2014). A most recent study using lentivirus and CD68 promoter to drive NeuroD1 overexpression in striatal microglia also converted them into neurons (Matsuda et al., 2019), whereas we previously used retrovirus and CAG promoter to express NeuroD1 but could not convert cortical microglia into neurons (Guo et al., 2014), raising an interesting question regarding different viruses and promoters in affecting *in vivo* cell conversion in different brain regions. Another surprising finding about NeuroD1 is that even in 14-month old Alzheimer’s disease mouse model (5xFAD), NeuroD1 can still efficiently convert reactive astrocytes into functional neurons, characterized by highly functional electrophysiological properties (Guo et al., 2014). Obtaining functional neurons in the adult brain is critical, because immature neurons often cannot survive well in the adult brain, and this is especially true for neurons in an injured or diseased environment (Buss et al., 2006). Generating a large number of functional new neurons in injured brains is the first step toward functional repair.

Different from the NeuroD1-mediated direct glia-to-neuron conversion, Zhang and colleagues reported successful neuroregeneration from astrocytes using a neural stem cell factor Sox2 to reprogram astrocytes back to neuroblasts and then further differentiate into neurons in both brain and spinal cord (Niu et al., 2013, 2015; Su et al., 2014; Islam et al., 2015; Wang et al., 2016). Importantly, these converted neurons are also electrophysiologically functional (Niu et al., 2013). The advantage of this approach is the potential of generating multiple neurons by reversing astrocytes back to neuroblast cells. The disadvantage is a two-step approach with additional factors needed for further neuronal differentiation such as BDNF and Noggin, VPA, or silencing p53. It is worth to note that the successful production of a significant number of mature and functional neurons in the adult spinal cord may provide a potential regeneration-based treatment for spinal cord injury. Further studies are warranted to generate subtype-specific neurons such as motor neurons to facilitate motor functional recovery after spinal cord injury.

The expression of Ascl1 was shown to convert astrocytes into functional glutamatergic and GABAergic neurons in dorsal midbrain, striatum, and somatosensory cortex of postnatal and adult mice *in vivo* (Liu et al., 2015). A combination of three transcription factors Ascl1, Lmx1a, and Nurr1 could turn striatal NG2 glia into mainly GABAergic neurons and a small proportion of glutamatergic neurons (Torper et al., 2015). Combining Ascl1 with Sox2 has been reported to transform NG2 glia into relatively immature neurons (mostly Doublecortin^+^) in the stab-injured cortex of adult mice *in vivo* (Heinrich et al., 2014). Ngn2 alone showed low conversion efficiency *in vivo*, but addition of Bcl-2 and anti-oxidative treatment (such as vitamin E and α-Tocotrienol) together with Ngn2 was reported to enhance conversion efficiency significantly, suggesting that oxidative stress may act as a major hurdle for cell conversion (Gascon et al., 2016).

It is worth noting that neurons not only can be reprogrammed from non-neuronal cells but also from other subtypes of neurons. For instance, overexpression of transcription factor Fezf2 can differentiate GABAergic progenitors in the mouse striatum into glutamatergic corticofugal projection neurons (Rouaux and Arlotta, 2010). Similarly, overexpression of Fezf2 in cortical layer IV neurons (De la Rossa et al., 2013) or layer II/III neurons (Rouaux and Arlotta, 2013) can change their identity into layer-V/VI corticofugal neurons. A recent study by Zhang and colleagues converted mouse striatal GABAergic neurons into dopaminergic neurons using a cocktail of factors including SOX2, Nurr1, Lmx1a, and Foxa2, and VPA (Niu et al., 2018). Changing one neuron subtype into another might cause unwanted outcome such as tilting the excitation-inhibition (E-I) balance (Ye et al., 2015). Because neurons cannot divide but glial cells can, it is ideal to use glial cells as conversion source instead of changing preexisting neurons.

### *In vivo* Reprogramming Glial Cells Into GABAergic Neurons

GABAergic interneurons (INs) (γ-aminobutyric acid secreting neurons), constituting around 10–20% of all neurons in the brain, are critical for balancing the excitatory and inhibitory tone in neural networks (Caputi et al., 2013). The breakdown of E-I balance causes epilepsy, depression, schizophrenia, and other neurological disorders (Caputi et al., 2013). Using various pro-neuronal transcriptional factors (TFs), GABAergic INs have been *in vivo* converted from different types of non-neuronal cells. We reported that about 10% of glia-converted neurons after ectopic expression of NeuroD1 in reactive glial cells were GABAergic neurons (Guo et al., 2014). Neurogenin-2 combined with growth factors (FGF2 and EGF) were able to covert proliferative non-neuronal cells residing in stab-injured rat striatum into mainly GABAergic neurons, but the total reprogramming efficiency was quite low (∼4%) (Grande et al., 2013). To increase the reprogramming efficiency, other TFs or TFs combined with small molecules were explored. Niu et al. (2015) found that transiently over-expressing Sox2 in the striatal resident astrocytes and subsequently treating with VPA could induce calretinin^+^ INs. Besides astrocytes, striatal NG2 glia cells could also be targeted. Ascl1, Lmx1a, and Nurr1 could successfully convert NG2 glial cells into GAD65/67+ neurons in intact striatum (Torper et al., 2015), and predominately fast-spiking parvalbumin^+^ (PV) neurons in the striatum (Pereira et al., 2017). In addition, oligodendrocytes in the striatum were reported to be reprogrammed into striatal neurons (Weinberg et al., 2017). The converted GABAergic neurons are functional, with mature electrophysiological firings and integrations (Grande et al., 2013; Niu et al., 2015; Pereira et al., 2017; Weinberg et al., 2017). However, compared with the diversity of GABAergic subtypes in the brain, the reprogrammed GABAergic neurons so far are very limited in subtypes. The conversion of GABAergic medium-spiny neurons (MSN, DARPP32+) and somatostatin expressing INs (SST+) are of less efficiency (Niu et al., 2015; Pereira et al., 2017). MSNs, the major projection neurons residing in the striatum and responsible for initiating and controlling movements, can be impaired when suffering from Huntington’s disease or stroke. SST neurons represent around 30% of the total IN population in many brain regions and their dysfunctions have been associated with seizure disorders (Urban-Ciecko and Barth, 2016). Thus, to generate specific GABAergic subtypes in specific brain areas under specific neurological disorders is a fundamental issue to be addressed for *in vivo* glia-to-neuron conversion approach.

### *In vivo* Reprogramming Glial Cells Into Dopaminergic Neurons

Parkinson’s disease (PD) is a common neurodegenerative disorder mainly caused by degeneration of dopaminergic neurons in the substantia nigra. Cell replacement therapy has been tested as a potential therapy to restore motor functions in PD patients. Researchers have provided evidence that dopaminergic neurons, derived from the human fetal ventral midbrain, are able to partially improve the clinical outcome in a few PD patients (Kefalopoulou et al., 2014; Li et al., 2016). But, there are still ethical concerns and technical difficulties in obtaining and standardizing human fetal tissue for transplantation, as well as the risk of graft-induced dyskinesia (Olanow et al., 2003; Politis et al., 2010). An alternative approach for generating dopaminergic neurons is to *trans*-differentiate glial cells into dopaminergic neurons directly inside the brains *in vivo*, eliminating the risk of external cell transplantation. Caiazzo et al. (2011) first reported in cell cultures that three transcription factors, Ascl1 (Mash1), Lmx1a/b, and Nurr1 (together referred as ALN) can reprogram mouse fibroblasts or glial cells into dopaminergic neurons *in vitro* (Addis et al., 2011; Torper et al., 2013). However, in mouse brain *in vivo*, the ALN combination fails to convert astrocytes or NG2 glia into TH^+^ dopaminergic neurons (Torper et al., 2015), but rather into GABAergic neurons. More recently, a study found that by combining three transcription factors, NeuroD1, Ascl1, and Lmx1a, together with microRNA 218, mouse astrocytes can be converted into dopaminergic neurons *in vivo* (Rivetti di Val Cervo et al., 2017), although the efficiency is relatively low (below 20%). Interestingly, a different combination of factors including SOX2, Nurr1, Lmx1a, and Foxa2, and VPA can convert striatal GABAergic neurons into dopaminergic neurons (Niu et al., 2018). More studies are necessary to find efficient conversion of glial cells into dopaminergic neurons for effective treatment of PD.

### *In vivo* Reprogramming Glial Cells Into Retinal Neurons

Unlike fish or chicken that can functionally regenerate their retina after injury (Fischer and Reh, 2001; Nagashima et al., 2013; Lenkowski and Raymond, 2014; Gallina et al., 2016), the adult mammalian retina has a restricted ability for self-regeneration. Previous work showed that Ascl1 is a key factor involved in Müller glia (MG) de-differentiation and retina regeneration in the fish (Fausett et al., 2008; Ramachandran et al., 2010, 2012; Wan et al., 2012). Pollak et al. (2013) further demonstrated that forced expression of Ascl1 in mouse MG induces a neurogenic state in *in vitro* condition. In mouse retina *in vivo*, overexpression of Ascl1 alone can only convert MG into immature neurons in young mice (<1 month old) with NMDA (*N*-methyl-D-aspartate) damage (Ueki et al., 2015). After combining Ascl1 with the histone deacetylase inhibitor trichostatin-A (TSA), Müller glia in adult mice with NMDA damage can be converted into more mature inner retinal neurons, including bipolar cells and amacrine cells, but rarely ganglion neurons (Jorstad et al., 2017). A different approach is to convert adult rods into cones, via knockdown of the rod photoreceptor determinant Nrl or Nr2e3, which appeared to preserve retinal tissue and restore visual function (Montana et al., 2013; Zhu et al., 2017). Most recently, Yao et al. (2018) reported that following gene transfer of β-catenin, cell-cycle-reactivated MG can be reprogrammed *in vivo* to generate new rod photoreceptors that integrate into retinal circuits and restore vision in mice by subsequent gene transfer of transcription factors Otx2, Crx, and Nrl. The next challenging task is to regenerate functional ganglion neurons in the adult mammalian retina.

To help the readers quickly grasp the rapid advancement of the emerging new field of engineered adult neurogenesis through glia-to-neuron conversion, we have compiled a comprehensive list of studies reporting on adult neurogenesis via *in vivo* reprogramming (Table 1). It sums up how different subtypes of neurons can be generated from different subtypes of glial cells from different brain regions through different transcription factors and compound treatments.

**Table 1:** Different subtypes of neurons generated from glial cells via *in vivo* reprogramming.

Neuronal subtype	Brain region/species	Glia source	Transcription factor	Reference
Glutamatergic	Cortex/mouse	Astrocytes	NeuroD1	Guo et al., 2014
Glutamatergic	Cortex/rat	?	Ngn2 + FGF2 + EGF	Grande et al., 2013
Glutamatergic	Cortex/mouse	Astrocytes	Ngn2 + Bcl2 + α-Tocotrienol	Gascon et al., 2016
Glutamatergic/GABAergic	Cortex/mouse	NG2 cells	NeuroD1	Guo et al., 2014
GABAergic	Striatum/rat	?	Ngn2 + FGF2 + EGF	Grande et al., 2013
GABAergic	Striatum/mouse	Astrocytes	Ascl1 + Sox2 + VPA or BDNF-noggin	Niu et al., 2015
GABAergic	Striatum/mouse	NG2 cells	Ascl1 + Lmx1a + Nurr1	Pereira et al., 2017
GABAergic	Striatum/rat	Oligodendrocytes	Polypyrimidine tract-binding protein (−)	Weinberg et al., 2017
GABAergic/Glutamatergic	Midbrain/mouse	Astrocytes	Ascl1	Liu et al., 2015
GABAergic/Glutamatergic	Striatum/mouse	NG2 cells	Ascl1 + Lmx1a + Nurr1	Torper et al., 2015
Neuroblasts	Striatum/mouse	Astrocytes	Sox2	Niu et al., 2013; Islam et al., 2015
Neuroblasts	Striatum/mouse	Astrocytes	Ascl1 + Sox2	Niu et al., 2015
Neuroblasts	Spinal Cord/mouse	Astrocytes	Sox2 + P53 (−)	Su et al., 2014; Wang et al., 2016
Dopaminergic	Striatum/mouse	Astrocytes	NeuroD1 + Ascl1 + Lmx1a + miR218	Rivetti di Val Cervo et al., 2017
Immature retinal neurons and photoreceptors	Retina/mouse	Müller glias	Ascl1	Ueki et al., 2015
Retinal interneurons	Retina/mouse	Müller glias	Ascl1 + TSA	Jorstad et al., 2017
Cone photoreceptors	Retina/mouse	Rod photoreceptors	Nrl or Nr2e3 (−)	Montana et al., 2013; Zhu et al., 2017
Rod photoreceptors	Retina/mouse	Müller glias	Otx2 + Crx + Nrl + β-catenin	Yao et al., 2018
?	Striatum/mouse	Astrocytes	ASCL1 + Brn2 + Myt1	Torper et al., 2013
?	Cortex/mouse	NG2 cells	Sox2	Heinrich et al., 2014

*Different subtypes of reprogrammed neurons are color coded in the table. Question mark “?” indicates mixed or uncertain cell types, while minus sign in parentheses “(−)” suggests knockdown of a particular gene product.*

### Mechanisms of *in vivo* Glia-to-Neuron Conversion

The mechanisms that govern the *in vivo* reprogramming of non-neuron cells into neurons are mostly unknown and understudied, and the mechanisms for *in vitro* reprogramming may not be readily applicable to *in vivo* conditions, given multiple discrepancies between *in vitro* and *in vivo* reprogramming (Kim et al., 2003; Heinrich et al., 2010; Caiazzo et al., 2011; Grande et al., 2013; Torper et al., 2013, 2015; Gascon et al., 2016). In general, the master transcription factors such as NeuroD1, Sox2, Ngn2, and Ascl1 play a major role in guiding the glia-converted neuronal fate, but the intrinsic lineages of the glial cells and local environmental cues both contribute to the final cell fate of *in vivo* reprogramming (Berninger and Jessberger, 2016; Li and Chen, 2016). The forced expression of NeuroD1 converts reactive astrocytes and NG2 cells into neurons (Guo et al., 2014), and microglia into neurons as well (Matsuda et al., 2019), potentially through the activation of downstream neural transcription factors (Gao et al., 2009; Kuwabara et al., 2009; Boutin et al., 2010), epigenetic modulation (Matsuda et al., 2019), and regulation of the Sonic hedgehog (SHH) signaling pathway (Sirko et al., 2013). Sox2 may prime the epigenetic landscape (Amador-Arjona et al., 2015) and directly regulates Tlx enhancer activity during *in vivo* reprogramming of astrocytes (Islam et al., 2015). Interestingly, Ngn2-mediated *in vivo* reprogramming appears to require the co-expression of Bcl-2 to inhibit ferroptosis, and anti-oxidative treatments also improve Ngn2 conversion efficiency (Gascon et al., 2016). For Ascl1-induced conversion into neurons *in vivo*, one possible mechanism relies on the capacity of accessing closed chromatin by Ascl1, when these chromatin loci are in a specific histone modification state (Wapinski et al., 2013). Reprogramming of Müller glia in mouse retina provides more insight into the mechanisms for *in vivo* neuronal conversion. For example, reduced chromatin accessibility leads to loss of neurogenic capacity in mature Müller glia (Ueki et al., 2015), and histone deacetylase inhibitor allows more effective reprogramming by promoting accessibility at key gene loci in the Müller glia (Jorstad et al., 2017). The cell cycle of Müller glia can be reactivated by gene transfer of β-catenin, and then these Müller glia can be reprogrammed into rod photoreceptors (Yao et al., 2018).

Similar transcription factors might convert glial cells into different subtypes of neurons in different brain regions, likely because glial cells in different brain regions arise from different lineages during development. For instance, glial cells in cortex and striatum can be reprogrammed into glutamatergic and GABAergic neurons by Ngn2, respectively (Grande et al., 2013; Gascon et al., 2016). Likewise, Ascl1 can convert glial cells in the striatum and midbrain into GABAergic neurons, but it converts retina Müller glia into retinal neurons (Liu et al., 2015; Torper et al., 2015; Ueki et al., 2015; Jorstad et al., 2017; Pereira et al., 2017). The impact of the local microenvironment on *in vivo* reprogramming is also strong. For example, the adult neocortex and striatum respond differently to Ngn2 and growth factors and generate new neurons with distinct molecular phenotypes (Grande et al., 2013). Brain or spinal cord injury can promote neurogenesis by rendering cells more permissive to chromatin remodeling (Dimou and Gotz, 2014; Heinrich et al., 2014), or unleashing the neurogenic potential restrained by Notch signaling (Magnusson et al., 2014). Lastly, chemical reprogramming may act through epigenetic silencing of glial genes (e.g., *GFAP* and *ALDH1L1*) and transcriptional activation of neural transcription factors and neuron-enriched genes (e.g., NeuroD1, Neurogenin2, *MAP2*, and *NEUN*) (Zhang et al., 2015; Gao et al., 2017). Small-molecule treatment increases DNA methylation in the *GFAP* promoter region but decreases H3K27 methylation at the Ngn2 transcription start site, therefore promoting glia-to-neuron conversion (Zhang et al., 2015).

Endogenous adult neurogenesis is a natural process similar to embryonic neurogenesis, both of which involve NSC differentiation due to intrinsic transcriptional regulation. In contrast, *in vivo* reprogramming involves an engineered cell fate change through ectopic expression of neural transcription factors that are normally expressed very low in glial cells (Li and Chen, 2016). The intrinsic NSCs or NPCs are limited, whereas glial cells can provide unlimited cell source for *in vivo* reprogramming, because astrocytes and NG2 cells are present throughout the CNS and not as easily exhaustible as NSCs in adult animals (Li and Chen, 2016). On the other hand, for both adult neurogenesis and *in vivo* cell conversion, newborn neurons are surrounded by an adult environment that may be full of neuroinhibitory factors and immune cells, particularly after injury or diseases. Unless there are sufficient number of newborn neurons that can support each other and quickly establish synaptic connections with preexisting neurons, the newly generated neurons may not survive well (Gascon et al., 2016). Therefore, the total number of newborn neurons generated in an adult environment is critical for their long-term survival and functional integration.

### Advantages and Challenges of *in vivo* Glia-to-Neuron Conversion

Compared to *in vivo* cell conversion, *in vitro* engineered neurogenesis currently dominates the field of regenerative medicine, largely because stem cells are easy to culture in an *in vitro* environment than to manipulate in an adult animal. Past several decades have gained much insight into the molecular mechanisms of stem cell self-renewal and differentiation, including neural differentiation of embryonic stem cells and iPSCs. However, despite some success of cell transplantation in animal models showing symptomatic relief or even partial functional improvement, there still lacks a widely applicable stem cell therapy to treat severe neurological disorders in a disease-modifying manner. The potential reasons for this gap between basic research and clinical applications regarding *in vitro* engineered neurogenesis might include the efficacy of neural differentiation, the purity of differentiated neuronal subtypes, the potential of tumorigenesis associated with stem cells, the potential gene mutations accumulated during multi-generation subcultures, the potential immunorejection after transplantation, and ethical concerns associated with embryonic stem cells and human aborted fetal tissues. In contrast, all the cell culture-associated drawbacks are automatically overcome by *in vivo* cell conversion approach.

In addition, the engineered *in vivo* cell conversion technology also has a number of advantages over endogenous adult neurogenesis. Using endogenous glial cells that are nearby the lost neurons for regeneration is perhaps the most economical way to replenish the lost neurons and restore the lost functions. Because glial cells are widespread in the CNS, *in vivo* glia-to-neuron conversion technology can regenerate a large number of new neurons throughout the CNS, whereas the endogenous adult neurogenesis is rather limited in mammalian brains (Arvidsson et al., 2002; Sun et al., 2012; Magnusson et al., 2014; Tobin et al., 2014). Another potential advantage is that converting dividing reactive glial cells into non-dividing neurons not only reduces the number of reactive glial cells but also decreases the tumor risk associated with dividing glial cells. Therefore, we predict that a highly efficient glia-to-neuron conversion technology will be the next-generation therapy for brain and spinal cord repair.

Like any new groundbreaking technology, the *in vivo* glia-to-neuron conversion approach also faces many challenges ahead before potential translation into clinical therapies. One big challenge is how to convert local glial cells into neurons with identity similar to the regional neurons. This may require screening an optimal combination of transcription factors (TFs) that can mimic the early neuronal specification process. The recent advent of algorithms to predict TFs involved in cell-cell conversion might help to accelerate this process (Rackham et al., 2016; Ronquist et al., 2017). Another challenge is to convert glial cells into fully functional neurons and achieve long-term survival in the adult brains. Many published studies have shown successful glia-to-neuron conversion *in vivo*, but only a few studies demonstrate highly functional properties of the glia-converted neurons using electrophysiological recordings. The third challenge is to demonstrate the integration of newly converted neurons not only into the local neural circuits but also into the global neural circuits by performing anterograde and retrograde tracing experiments. One concern is whether the newly generated neurons might form wrong targets with neighboring or distant cells. This is certainly possible but according to the developmental principles of “wiring together, firing together; firing together, wiring together,” those wrongly formed connections might be eliminated if there is not enough experience-dependent activity to support their existence. Another concern is whether too many neurons might be generated through *in vivo* glia conversion? This is again not completely excludable, but a simple solution is to find a suitable dose of viral vectors so that neurons will not be overproduced. In fact, direct glia-to-neuron conversion has not been able to generate 100% of lost neurons to fully reverse the neuronal loss yet. Interestingly, we found that NeuroD1 conversion efficiency in reactive astrocytes is very high (90%) (Guo et al., 2014), but another group reported low efficiency in non-reactive astrocytes (Brulet et al., 2017), suggesting that reactive state might facilitate conversion. Similarly, Sox2 was found to convert reactive NG2 cells into neurons in injured adult cortex, but not resting NG2 cells without injury (Heinrich et al., 2014). From neural repair point of view, such high conversion efficiency in reactive glial cells but low efficiency in resting glial cells is ideal, because one may not want to disturb too much the resting glial cells. It is the reactive glial cells in the injured areas that are the best cell source for *in vivo* conversion. On the other hand, we also noticed that some reactive glial cells in the lesion core might be more difficult to convert than the reactive glial cells in the penumbra areas, suggesting that the degree of injury also affects conversion efficiency. Last but not least, we have never observed glial depletion in NeuroD1-converted areas even though its conversion efficiency is very high, because glial cells have intrinsic proliferation capability. This is very important because glial cells play important roles in supporting neuronal functions including promoting synapse formation and providing nutrients to neurons. It is important to note that *in vivo* cell conversion typically converts a subset of reactive glial cells into neurons, and the remaining glial cells can further divide to repopulate themselves to avoid being depleted.

Besides challenges, there are certainly some limitations of *in vivo* cell conversion. One obvious limitation is that when injury or degeneration is too severe, such as massive tissue loss at late stage, *in vivo* cell conversion might not be sufficient to generate enough cells. Perhaps grafting external cells or artificial tissue might be necessary to repair the tissue loss. Another limitation is that if neurodegeneration is caused by gene mutation, converted neurons would still carry the mutation and might still degenerate later. Although for neurodegenerative disorders, the newborn neurons would survive for a significant time before start to degenerate again, the ideal strategy may be to combine cell conversion together with gene editing technology to correct the mutation so that converted neurons can survive even longer. Finally, while most of the neural transcription factors used for *in vivo* cell conversion, such as NeuroD1, Ngn2, and Ascl1, are endogenous proteins expressed in neurons during brain development, the potential impact of their constitutive expression in adult neurons needs to be fully assessed.

The advantage of overexpressing TFs through viral vectors is that it can be precisely delivered into the brain or spinal cord where injury occurs. However, such precision viral delivery requires high technical skill of neurosurgeons and well-equipped hospitals. For rural areas with scarce medical facilities, gene therapy will be difficult to carry out. To overcome such limitation of gene therapy, we have tested the possibility of drug therapy and achieved the first step of success. Using a combination of small molecules, we are capable to transform cultured human astrocytes into functional glutamatergic neurons, at least in *in vitro* condition (Zhang et al., 2015). Following our success, Gao et al. (2017) also reported that human adult astrocytes isolated from glioma patients can be chemically converted into glutamatergic neurons in cell cultures. Our most recent study has successfully achieved chemical conversion of human astrocytes into neurons using 3–4 small molecules, making it one step closer for drug development (Yin et al., 2019). These studies paved the way toward future drug therapy, but *in vivo* studies on direct chemical reprogramming and efficient drug delivery across blood–brain-barrier are yet to be achieved before potential clinical translations. Nevertheless, it is worth the great effort to develop such drug therapy that can regenerate new neurons in patients’ brains not only to slow down the progression but also to reverse the course of neurodegenerative disorders.

## Author Contributions

WLe, WLi, LG, and GC contributed toward writing the review article. WLe and GC finalized the manuscript.

## Conflict of Interest Statement

GC is a founder of NeuExcell Therapeutics Inc. The remaining authors declare that the research was conducted in the absence of any commercial or financial relationships that could be construed as a potential conflict of interest.
